# Evolutionary Design of Convolutional Neural Networks for Human Activity Recognition in Sensor-Rich Environments

**DOI:** 10.3390/s18041288

**Published:** 2018-04-23

**Authors:** Alejandro Baldominos, Yago Saez, Pedro Isasi

**Affiliations:** Computer Science Department, Universidad Carlos III de Madrid, 28911 Leganes, Spain; yago.saez@uc3m.es (Y.S.); isasi@ia.uc3m.es (P.I.)

**Keywords:** neuroevolution, deep learning, convolutional neural networks, human activity recognition

## Abstract

Human activity recognition is a challenging problem for context-aware systems and applications. It is gaining interest due to the ubiquity of different sensor sources, wearable smart objects, ambient sensors, etc. This task is usually approached as a supervised machine learning problem, where a label is to be predicted given some input data, such as the signals retrieved from different sensors. For tackling the human activity recognition problem in sensor network environments, in this paper we propose the use of deep learning (convolutional neural networks) to perform activity recognition using the publicly available OPPORTUNITY dataset. Instead of manually choosing a suitable topology, we will let an evolutionary algorithm design the optimal topology in order to maximize the classification F1 score. After that, we will also explore the performance of committees of the models resulting from the evolutionary process. Results analysis indicates that the proposed model was able to perform activity recognition within a heterogeneous sensor network environment, achieving very high accuracies when tested with new sensor data. Based on all conducted experiments, the proposed neuroevolutionary system has proved to be able to systematically find a classification model which is capable of outperforming previous results reported in the state-of-the-art, showing that this approach is useful and improves upon previously manually-designed architectures.

## 1. Introduction

Internet of Things smart devices are currently experiencing unprecedented growth, offering a new range of services and solutions. These devices form large and heterogeneous sensor networks where context-aware systems are commonly needed for different reasons. Electronics, software, sensors, actuators, and wireless technologies enable these objects to connect and exchange data, which combined with artificial intelligence and machine learning systems open a completely unexplored horizon of novel applications and services.

Wireless sensor networks can be widely used to bring together and exchange environmental information from homes, buildings, vehicles, etc., where one of the most important and common challenges is performing an appropriate context identification. These context-awareness processes can be useful for many different tasks, for example providing a more customized experience and improved interaction to users. In this sense, with a proper context identification, health apps could monitor users’ behaviors in their daily lives, preventing harmful or unhealthy practices such as smoking or lack of physical exercise, and reward appropriate behaviors for their particular health status.

In correlation to the increasing number of sensors, there is also a rise of open data repositories, many of them related to smart cities [1,2]. Some of these datasets have been released to the public with information about human activity. An interesting example is the OPPORTUNITY dataset that is used in this work. This dataset collects different activities performed by subjects, labelled and correlated with the state of different sensors, both placed on the subjects’ bodies and in the surrounding environment. In other works, sensor networks collect environmental data together for context-aware services which can be used in gerontechnology for real-time patient tracking [3].

In this paper, we will approach this problem of human activity recognition using deep learning techniques, more specifically, a technique known as “convolutional neural networks” (CNNs). The advantage of using CNNs over classical machine learning techniques is that the former often require little to no preprocessing of the input data. In other words, whereas classical techniques often require to the signals to be transformed (e.g., computing the discrete Fourier transform), segmented, and have the manually-engineered features extracted, deep learning techniques are expected to learn relevant features automatically, and are able to work with raw data or data with almost no preprocessing. This technique has previously been applied to human activity recognition with the OPPORTUNITY dataset in works such as those by Ordóñez and Roggen [4] or Hammerla et al. [5]. On the other hand, the biggest drawback of the use of CNNs lies in the great impact that the design of the network topology has on the results obtained. This dependence makes it difficult to obtain good solutions due to the huge number of possible CNN designs, and the lack of an established analytical procedure for determining the optimal design will often lead us to waste time and end up building suboptimal solutions after significant amounts of trial-and-error. In this work, the topology of the CNN is not designed by hand; conversely, it is automatically inferred by an evolutionary algorithm which searches for the optimal network that maximizes the success rate over a validation set.

However, the evolution of CNNs faces a series of hardships. The training of convolutional networks is especially expensive, mainly due to the size and complexity of the architectures, as well as the need to use a large number of examples to achieve appropriate performances. This work tackles these issues by means of a particular neuroevolutionary procedure developed by the authors [6] and based on grammatical evolution (GE) that allows the evaluation of a sufficient number of individuals to obtain architectures that rival those designed by experts. Furthermore, the proposed method is able to generate a set of enough diverse individuals to use them in an ensemble paradigm, therefore improving the results. In addition, we want to verify the effectiveness of the new method in the design of CNN models capable of offering competitive results in the detection of human activity when signals from different sensors are used.

This paper is structured as follows: Section 2 presents some theoretical foundations required to understand convolutional neural networks, and Section 3 discusses related works regarding the neuroevolution of CNNs. Then, Section 4 describes the OPPORTUNITY dataset, a publicly available database which contains data gathered from opportunistically placed sensors and labelled with human activity information, and discusses previous works that have performed classification using this dataset. Later, in Section 5 the methods are described, explaining how a basic preprocessing of the input data is carried out and how the neuroevolutionary process is adapted in order to be applied to the OPPORTUNITY dataset. The results are shown and discussed in Section 6, and finally Section 7 provides some conclusive remarks and suggests future lines of work.

## 2. Convolutional Neural Networks

In this section, we will explain some concepts which are essential to understand the technical background of this paper. A important set of theoretical foundations are those related to CNNs, the machine learning technique used to perform activity recognition in this work.

CNNs combine a feature learning module inspired by the visual cortex of mammals with a trainable classifier such as a fully connected neural network. They were first introduced by LeCun et al. in 1998 [7,8]. The feature learning module is designed in order to replace a feature engineering stage which is often performed by hand. Thus, CNNs are suitable for receiving raw data as an input, and after features are learned by the convolutional layers, they will be passed through the classifier. CNNs provide invariance to local distortions of the input, and in order to extract features from the input data, they remain aware of the input topology and structure.

**Figure 1:**
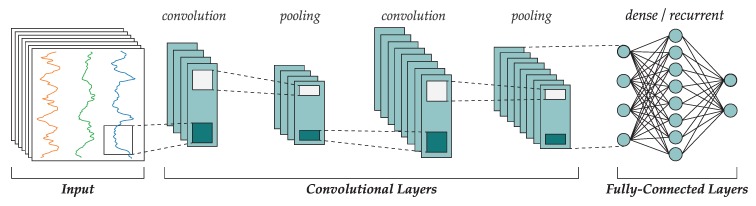
Common topology of a convolutional neural network.

Figure 1 shows the topology of a typical sequential CNN. First, the input data is passed to a convolutional layer, which will output certain features. The output of a convolutional layer will be introduced as input to the following layer, and in some cases there may be a pooling operator that reduces the dimensionality of the features tensor. Finally, after all convolutional layers have processed the input, the resulting tensor is flattened to a vector and introduced to a fully connected network (such as a multi-layer perceptron or a recurrent neural network).

### 2.1. Convolutional Layers

Convolutional layers are responsible for automatically learning features from input data. To do so, training data will be inputted to the first convolutional layer, which will compute and output *feature maps* that will be introduced to the next layer. This step is repeated throughout all the convolutional layers. Because the second and subsequent layers operate over the output of the previous layer, the larger the number of convolutional layers, the more abstract features the network will be able to learn.

**Figure 2:**
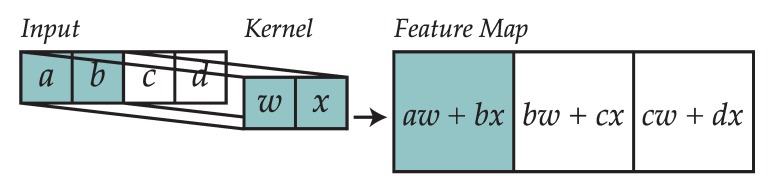
Example of how a 1D kernel is used to convolve the input.

Each layer is composed of kernels (also known as patches), which convolve the input generating as output a feature map. Both the input and the kernels are represented by means of multidimensional arrays, known as tensors. Figure 2 shows an example of how a kernel convolves the input to generate a feature map.

After computing feature maps, an activation function can be applied to every element in the output tensor. A common approach which is also implemented in this work is to apply a ReLU (rectified linear unit) function in order to compute a non-linear transformation of the output.

### 2.2. Pooling

After each convolutional layer, a pooling operator can be introduced to reduce the dimensions of the input by performing downsampling. The most widely used technique is known as *max-pooling*, where a subtensor of the input is replaced by its maximum value. The pooling operator is characterized by its size, which defines how large the subtensor will be: the larger the pooling size, the smaller the output.

Pooling has also been shown to introduce invariance to translation, thus potentially improving the robustness of the network to small changes in the input.

### 2.3. Dense and Recurrent Layers

After convolutional layers have extracted relevant features from raw data, these features can be introduced to a classifier, which in most cases will consist of a multi-layer perceptron or a recurrent neural network. In order to introduce the output tensor to the classifier, it must be flattened beforehand; i.e., turned into a vector.

The classifier network will be composed of several layers, each one comprising neurons or units, which will process the input in order to generate an output. A simple approach is that followed by feed-forward layers, where only the input from the previous layer is considered. However, when dealing with time series (such as those involved in this work, which are signals retrieved from different sensors across time), a recurrent neural network might lead to better classification performance. A simple version of a recurrent layer will involve connections from other neurons in the same layer, where the value in the previous iteration is taken into consideration.

In practice, these “vanilla” recurrent layers do not work properly, since backpropagation fails to accurately update the weights when the context goes long into the past. To solve this issue, LSTMs (standing for long-short term memory) were introduced by Hochreiter and Schmidhuber in 1997 [9]. An interesting variation of LSTM is called GRU (gated recurrent unit), introduced by Cho et al. in 2014 [10], resulting in a simpler unit, which is sometimes preferred to LSTM.

Finally, a regularization term can be introduced in each layer’s weights to prevent overfitting. Common approaches for regularization involve the addition in the loss function of either L1 (lasso) or L2 (ridge regression) norms. Additionally, a more recent technique called “dropout”, consisting of removing a random set of connections during training, has been proven successful [11] for dealing with overfitting.

### 2.4. Learning Rules

After determining the topology of the convolutional neural network, the backpropagation process can start to learn the network parameters. To achieve this training, “minibatches” of data from the training set will be introduced to the first convolutional layer, and an output is generated by the network, which is used to be compared with the expected output in order to compute a loss function. A “learning rule” (sometimes called “optimizer”) will update the weights in the direction of the gradient so that the value of this loss function reduces as training occurs.

A common learning rule is stochastic gradient descent (SGD), which slightly updates weights in the direction of the gradient. A momentum, also known as Nesterov momentum, can be introduced to control the velocity at which weights are updated. Additionally, some learning rules automatically scale the learning rate during training, such as AdaGrad [12], AdaDelta [13], or RMSProp [14]. More recently, Adam and Adamax [15] have been proposed claiming to combine some of the advantages of AdaGrad and RMSProp.

## 3. Related Work in Neuroevolution

After the AI Winter came to an end by the mid-1980s, neural networks reemerged to solve a great variety of problems. However, their use requires designing their topology, a decision with a potentially high impact on performance. Whereas many works have tried to suggest rules-of-thumb for designing topologies, the truth is that there are no analytical procedures for determining the optimal one, and trial-and-error is often used instead.

Neuroevolution is the name given to a field of computer science that applies evolutionary computation to evolve some aspects of neural networks. In particular, evolutionary algorithms are a set of search techniques that are able to evolve a population of individuals (which are identified by a genotype and can be translated to a phenotype) in order to optimize a certain metric throughout generations, known as fitness. In neuroevolution, the phenotype is a certain definition of some aspects of a neural network (e.g., its topology), and the genotype will depend on the technique used at each moment. Neuroevolution arose by the end of the 1980s, and has led to remarkable developments for evolving neural networks, such as EPNet [16], NEAT [17], or EANT [18], to mention some with the most impact.

While neuroevolution is a well-established research area, its application to convolutional neural networks is still marginal. This is because, as we have seen in the previous section, CNNs can involve very complex topologies, with dozens of convolutional layers comprising diverse depths and filter sizes, and with several dense or recurrent layers with a variable number of neurons and diverse activation functions. Therefore, only in recent years have improvements in computational resources (e.g., a greater availability of deep-learning-specific graphics processing units, GPUs) and research advances enabled the development and validation of tools to specifically optimize this type of deep neural network. Moreover, since the manual design of CNN topologies is expensive, neuroevolution is a promising approach to obtain optimal topologies for a given problem.

To the best of our knowledge, and also according to the authors themselves, the first work in this field was presented by Koutník et al. [19] in 2014, who did not evolve the network topology but rather encoded the weights of a fixed architecture involving four convolutional layers with max-pooling and a simple recurrent network with few hidden units.

Later, in 2015, Verbancsics and Harguess [20] proposed a variation of HyperNEAT [21] (which at the same time is based on NEAT) to support the evolution of CNNs. Another work published that year by Young et al. [22] proposed MENNDL, an approach which used a genetic algorithm (GA) to evolve the hyperparameters of a neural network. However, the latter focused on a very narrow search space since it involved only six hyperparameters: the number of kernels and the kernel size for a CNN with three layers.

In 2016, Loshchilov and Hutter [23] proposed the use of CMA-ES (covariance matrix adaption evolution strategy) to evolve some hyperparameters of deep neural networks, such as number of kernels, number of hidden units in dense layers, dropout regularization, batch size, or other learning hyperparameters. Their approach considers a larger search space than the previous work by Young et al., although some aspects of the convolutional layers (kernel sizes, activation functions, etc.) remained out of the scope of the evolutionary process. Another work in 2016 by Fernando et al. [24] suggested the use of a differentiable version of a CPPN (compositional pattern producing network, a variation of a neural network introduced in NEAT to ease evolution) called DPPN, which could ultimately replicate CNN topologies.

Most works involving further development in the neuroevolution of deep learning networks arose in 2017. For example, Xie and Yuille [25] presented GeNet, where a GA evolved CNN topologies under certain constraints (e.g., a limited number of layers) using predefined building blocks, such as convolution or pooling. However, GeNet does not focus on evolving the hyperparameters of fully-connected or recurrent layers. Another example is CoDeepNEAT by Miikkulainen et al. [26], which follows the same principles as NEAT, allowing learning very complex networks with different convolutional or dense setups (including recurrent layers), although it strongly relies on mutation.

Desell [27] introduced EXACT in 2017, which largely focused on describing how the neuroevolution system was supported by a volunteer computing architecture. EXACT does not implement the logic for evolving pooling operators, activation functions, dense layers, or some backpropagation hyperparameters (such as the learning rate). A similar approach has been presented by Real et al. [28], from the Google Brain team, with the additional advantage that authors tested the performance of ensembles of evolved models. Additionally, Davison created an open-source tool named DEvol, using genetic programming, to evolve the number of layers, the number of kernels, the dropout rate, the activation function, etc. Suganuma et al. [29] used a variation of genetic programming known as Cartesian genetic programming to evolve some aspects of convolutional layers, although their approach does not consider dense layers or optimization hyperparameters.

Besides neuroevolution itself, some works have proposed the use of other techniques. Remarkable contributions include MetaQNN from Baker et al. [30], which uses reinforcement learning to search for optimal CNN topologies within the space of network architectures, and the training task is performed by sequentially choosing neural network layers. It is noticeable that MetaQNN does not optimize hyperparameters such as the learning rate or the batch size, does not include recurrent layers as possible states to be included in the architecture, and does not optimize the neurons’ activation functions. A similar work, including recurrent connections, has recently been published by Zoph and Le [31] from the Google Brain team.

The automatic design of CNN topologies is a trend shifting from academic works to industry; for example, Google released AutoML [32] to—among other features—determine suitable CNN topologies in Google Cloud, and BigML has also recently introduced Network Search [33] for the Deepnets product in their cloud.

The neuroevolutionary approach presented in this paper incorporates some of the features that can be found in related works, but focusing on a very broad number of aspects to be optimized, including a variable number of convolutional and fully-connected or recurrent layers, activation functions, or learning hyperparameters. To enhance the flexibility of individuals to be evolved, we will use grammatical evolution (GE), which allows using an encoding with low redundancy and is very flexible. Additionally, we have combined several individuals found after the evolution is completed in order to build ensembles, which translates into an improvement of the performance.

Table 1 shows a brief comparison of the system proposed in this paper against related works. The abbreviations shown in the table header stand for the next criteria:

- **Var. Ly.**: whether the proposal supports a variable number of layers (either convolutional, fully-connected, recurrent, etc.).
- **Conv.**: whether the proposal evolves the convolutional layers or some of their parameters.
- **FC**: whether the proposal evolves fully-connected layers or some of their parameters.
- **Rec.**: whether the proposal observes the inclusion of recurrent layers or LSTM cells.
- **Act. Fn.**: whether the proposal evolves the activation function instead of hardcoding it.
- **Opt. HP**: whether the proposal supports the evolution of optimization hyperparameters (learning rate, momentum, batch size, etc.).
- **Ens.**: whether the proposal supports the construction of an ensemble of neural networks.
- **W**: whether the proposal evolves the weights of the network.

## 4. Human Activity Recognition

To test the ability of the model to classify different types of human activities, we decided to use the OPPORTUNITY dataset as a testbed. In this section, we will describe this dataset, explaining how the data were obtained (according to the specifications provided by the authors) and the performance obtained by other researchers using this dataset, which can serve as a benchmark to evaluate our proposal.

### 4.1. OPPORTUNITY Dataset

OPPORTUNITY is a complex human activity dataset introduced by Roggen et al. in 2010 [35]. This dataset results from an EU-funded project whose aim is to recognize human activity from *opportunistically* discovered sensors (thus the name). Opportunistic sensor configurations imply that sensors are not placed on the body at precise locations. In contrast, sensors which are already present are used, even when their number, placement, and setup are not known in advance.

According to the authors, OPPORTUNITY data were recorded in a sensor rich environment consisting of “*a room simulating a studio flat with kitchen, deckchair, and outdoor access where subjects performed daily morning activities*” [35]. Seventy-two sensors belonging to 15 networked sensor systems and comprising 10 modalities were deployed over the environment, the objects, and the subject’s body.

The purpose of the OPPORTUNITY dataset is to record daily human activities in a realistic manner, trying to keep their execution natural. The authors did not provide specific instructions to subjects on how to perform the activities, thus leaving free interpretation and encouraging them to perform the tasks in the usual way they would do. Authors configured a sensor-rich environment, an approach which poses some benefits: activities are sensed by multiple sensors, sensors in close proximity provide robustness against sensor placement variability, and reading sensors from diverse modalities and/or systems allows their performance or reliability to be assessed.

In particular, six sensor systems were deployed on the subjects’ bodies, one sensor system was placed on the objects, and eight sensor systems were deployed in the environment. Table 2 describes the different systems as provided by authors in the original OPPORTUNITY paper [35].

The fact that many wireless sensors are closely placed also introduced additional technical challenges when building a suitable sensing environment. Because they used some proprietary sensor systems along with custom devices, different systems were required for the data acquisition infrastructure. In particular, seven computers (six laptops and a desktop PC) were used with diverse proprietary software along with the CRN Toolbox previously introduced by Bannach et al. [42]. These computers were provisioned according to the needs of the sensors systems (e.g., video and audio streams required a dedicated computer, some sensors required a computer to be placed within a range, etc.). Room lighting was controlled by closing the blinds to avoid sunlight changes and switching on fluorescent tubes for the whole experiment (except when subjects were required to turn them off as a part of the protocol).

During the experiment, subjects were asked to perform five ADL (activity of daily living) runs and one drill run. The ADL runs comprised the next sequence of activities:

1. Start: lie in the deckchair and then get up.
2. Groom: move across the room, checking that objects are in the right drawers and shelves.
3. Relax: go outside and walk around the building.
4. Prepare coffee: use the coffeemaker to prepare coffee with milk (in the fridge) and sugar.
5. Drink coffee: take coffee sips naturally.
6. Prepare sandwich: made of bread, cheese and salami and using the bread cutter along with various knifes and plates.
7. Eat the sandwich.
8. Cleanup: clean the table, store objects back in their place or in the dish washer.
9. Break: lie on the deckchair.

Activities in the ADL run can be considered with different levels of abstraction, thus enabling a hierarchy of actions. For example, an abstract activity could be “*prepare a sandwich*”, comprising some composite activities such as “*cut bread*”, which at the same time can be composed of atomic activities like “*move to bread*” or “*operate bread cutter*”. Each ADL lasted about 15–25 min, and breaks of 10–20 min between runs were given to copy data and ensure that devices were charged and running properly. An instructor guided subjects during the first run, placing very few constraints. Thus, subjects were asked to follow the high-level action sequence, and allowed to interleave actions (e.g., prepare the sandwich while still drinking coffee), switch hands, etc.

After the five runs, subjects had to complete the drill run, where they were asked to perform 20 repetitions of the following sequence, in order to generate many activity instances:

1. Open and close the fridge.
2. Open and close the dishwasher.
3. Open and close 3 drawers at different heights.
4. Open and close door 1.
5. Open and close door 2.
6. Turn on and off the lights.
7. Clean table.
8. Drink while standing.
9. Drink while sitting.

Drill runs lasted between 20 and 35 min. After all the experiments took place, an open source tool was used to annotate the dataset. This annotation was done in four different tracks: the first contains modes of locomotion (sitting, standing, walking, etc.), the following two contain actions of the left and right hand (reach, grasp, release, etc.) along with the object of the interaction, and the last contains high-level activities (prepare sandwich, prepare coffee, etc.). According to authors, 30 min of data recording required between 7 and 10 h of annotation time.

The reader can find a much more detailed description of the dataset in Deliverable D5.1 of the OPPORTUNITY Project [43], including maps showing the placement of sensors, technical specifications of the sensing devices, and signal processing techniques used to compose the final dataset.

### 4.2. OPPORTUNITY Challenge and State-of-the-Art Results

A subset of the OPPORTUNITY dataset was used for the OPPORTUNITY Activity Recognition Challenge which took place in the Workshop on Robust Machine Learning Techniques for Human Activity Recognition of the 2011 IEEE Conference on Systems, Man and Cybernetics [44,45]. This subset finally comprised 4 subjects and 113 sensor channels grouped in two different datasets: the locomotion dataset and the gestures dataset.

The locomotion dataset includes four classes: *stand* (1093), *sit* (1095), *walk* (90), and *lie* (40). The number between parentheses indicates the number of instances associated to that label. The gestures dataset includes 17 classes: *open dishwasher* (50), *close dishwasher* (56), *open fridge* (129), *close fridge* (133), *open drawer 1* (50), *close drawer 1* (49), *open drawer 2* (44), *close drawer 2* (44), *open drawer 3* (56), *close drawer 3* (57), *open door 1* (45), *close door 1* (39), *open door 2* (43), *close door 2* (41), *move cup* (184), and *clean table* (33). Additionally, instances in both datasets may not be labelled (i.e., belong to a “*null*” class), and this *null* class has a high prevalence. Numbers show that the first dataset is very unbalanced (most of the times subjects are sitting or standing), whereas the second is mostly balanced, except for the “*move cup*” class.

In this section, we will report performance in terms of the weighted F1 score metric, whose formula is shown in Equation (1), with $n_{c}/N$ being the proportion of samples of class *c*:

(1) $$F_{1}=2\sum_{c}\left(\frac{n_{c}}{N}\frac{precision_{c}\times recall_{c}}{precision_{c}+recall_{c}}\right)$$

We will adhere to the guidelines provided in the OPPORTUNITY challenge for training and testing classifiers, in order to enable a side-by-side comparison:

- Training set: comprises all ADL and drill sessions for subject 1 and ADL1, ADL2, and drill sessions for subjects 2 and 3.
- Test set: comprises ADL4 and ADL5 for subjects 2 and 3.

When results were not available for the whole test set, then the average weighted F1 score for individual subjects 2 and 3 was computed.

A benchmark of different classification techniques was first published by the dataset authors for the workshop where the OPPORTUNITY challenge took place [46]. In the gestures dataset, best results were achieved using *k*-nearest neighbors (*k*-NN) with k=3, leading to an F1 score of 0.85 when considering the *null* class (which drops to 0.56 when the *null* class is removed). 3-NN also outperformed other classifiers in the locomotion dataset, where F1 scores ranging from 0.85 to 0.86 were attained regardless of whether the *null* class was considered or not.

In 2012, Cao et al. [47] proposed an integrated framework for human activity recognition, whose performance was tested with the OPPORTUNITY dataset. This framework performed an F1 score of up to 0.821 for the gesture recognition problem with *null* class, and 0.927 for locomotion recognition without *null* instances.

Additionally, a benchmark was published after the challenge took place, revising the baseline results and summarizing the most remarkable contributions [45]. In the case of the gesture recognition problem, an F1 score of 0.88 was achieved including the *null* class, dropping to 0.77 when the *null* instances were removed. In both cases, the best classifier was a combination of support vector machines (SVMs) with 1-nearest neighbor. Meanwhile, in locomotion recognition, the best performance was attained with *k*-NN when considering the *null* class (F1 score of 0.85) and decision tree grafting [48] when ignoring the *null* class (F1 score of 0.87).

In 2015, Yang et al. [49] proposed the first application of deep learning techniques to learn a classifier for the OPPORTUNITY dataset, yet only focusing on the gesture recognition problem and considering the *null* class, for which they report an average F1 score of 0.818. Results were slightly higher (up to 0.822) when processing data using a technique known as *smoothing*, which was formerly described by Cao et al. [47].

Early in 2016, Ordóñez and Roggen proposed *DeepConvLSTM* [4], a deep learning technique combining convolutional layers with LSTM cells. In this approach, they pass the input data through four convolutional layers with rectified linear units (ReLUs), and then through two recurrent layers with LSTM cells using a hyperbolic tangent activation function. Finally, the output is introduced to a softmax classifier. Their architecture features an F1 score of 0.915 in the gestures dataset, dropping to 0.866 when the *null* instances were removed. LSTM layers were proved to improve the performance of non-recurrent convolutional networks, which attained F1 scores of 0.883 and 0.783, respectively. Regarding locomotion recognition, Ordóñez and Roggen attained F1 scores of 0.895 and 0.93 (with and without *null* class, respectively) using *DeepConvLSTM*, and 0.878 and 0.912 using non-recurrent convolutional neural networks.

In another 2016 work by Hammerla, Halloran, and Plötz, they compared different deep learning solutions on the gesture recognition track of OPPORTUNITY with *null* instances [5]. They report an outstanding F1 score of 0.927 using bi-directional LSTM networks on a sample-by-sample basis.

Table 3 provides an extensive yet comprehensive side-by-side comparison of the most relevant state-of-the-art results attained for OPPORTUNITY, including both the locomotion and gesture recognition tracks with and without *null* instances when available. The upper side of the table shows the performance of classical machine learning approaches, while the lower side displays the results of deep learning techniques, with the latter often showing better results. The best results are highlighted in boldface. The symbol † near some values means that authors reported the performance on a subject-per-subject basis, and are the outcome of averaging the F1 score for subjects 2 and 3; as a result, those values are only indicative. Techniques are named as in the original papers, and the reader is referred to those papers to understand the specific setup for each technique.

## 5. Neuroevolution for Human Activity Recognition

In this section, we explain the process followed in order to learn a human activity recognition model given the raw data available in the OPPORTUNITY dataset. This process involves two steps: first, some basic preprocessing of the sensors data is carried out, and then the neuroevolutionary process is run in order to determine the most suitable CNN topology and learn an appropriate model which succeeds at classification.

### 5.1. Preprocessing

Very little preprocessing was performed before feeding the OPPORTUNITY data to the deep neural network. The main aim of this stage is to clean data to remove missing values present in the raw data, and to transform data into a format supported by a tensor processing library (e.g., TensorFlow or Theano).

The first step involves removing features which must be ignored for the OPPORTUNITY challenge. These features are quaternion coefficients obtained from the inertial measurement units (a total of 16 features) and features obtained from sensors placed in objects and in the environment; that is, only sensors placed in the subjects’ bodies were considered (removing another 119 features). The filtered dataset will contain 113 features (channels) plus the class.

Later, we perform linear interpolation in each channel in order to provide an estimation for missing values (most of them arising from Bluetooth sensors disconnecting during the recording). Then, if there are missing values left, we set them to zero.

Finally, data is normalized in the interval [0,1] in order to avoid saturation of the neural network. This normalization is performed following Equation (2), where $x_{c}$ refers to a sample in channel *c*, and $\hat{x_{c}}$ is the normalized value:

(2) $$\hat{x_{c}}=\frac{x_{c}-\min\left(c\right)}{\max(c)-\min(c)}$$

The data fed to the input layer of the deep neural network is shaped as a four-dimensional tensor with dimensions B×1×w×113, where *B* is the batch size (as we are using mini-batching to train the model) and *w* is the window size. It must be noted that this window is extracted from a sliding window over the input data, with size *w* and step $w_{step}$. To obtain the overlapping sliding windows efficiently, we followed the implementation suggested by Vinyard [50].

### 5.2. Grammatical Evolution of CNNs

In order to determine the optimal topology for the convolutional neural network that will constitute the human activity classifier, we will adhere to the neuroevolutionary procedure that was previously presented by Baldominos et al. [6].

The number of parameters involved in the design of CNNs is certainly very large according to criteria of feasibility and effectiveness, and includes, among others, the setup of each convolutional layer (number of kernels, kernel size, whether or not to perform pooling, etc.), each fully-connected layer (whether it is feed-forward or uses a recurrent implementation, the number of hidden units, whether regularization is performed, the activation function, etc.), and the optimization hyperparameters. Because the topology is the key design decision of a CNN, a large number of design alternatives were included for both convolutional and dense layers. In addition, different configurations of the inputs are taken into account, as well as the optimization hyperparameters that are considered as essential.

As CNN topologies can present very diverse structures with variable number of layers, each with different sizes and different topologies, it has been considered that a suitable evolutionary method would be grammatical evolution (GE) [51]. The use of grammars provides a simple mechanism for describing complex structures. In addition, grammars are flexible, intuitive, and easy to adapt to the specific characteristics of the problem to be solved. As a starting point, GE takes a grammar defined in Backus–Naur Form (BNF). The individuals of the population constitute the production rules that will be applied sequentially until generating a sentence of the language defined by the grammar—in the case that concerns us, a specific design of a CNN topology.

Unlike other evolutionary algorithms, in grammatical evolution the encoding is enforced by the technique itself. In particular, individuals in GE are encoded as a vector of integer numbers, also called *codons*. The researcher does not have to make any further assumptions about the encoding, and only has to make two decisions: the codon size (the maximum value an integer in the vector can take) and the maximum chromosome length.

Additionally, in GE, the method for decoding the genotype into a phenotype is already given by the technique: the developer must provide a formal grammar that is used by the mapping function in order to generate valid phenotypes from the genotype. In particular, the formal grammar will generate a language, such that the set of words in that language is the set of valid phenotypes. It is worth noting that a language can be potentially infinite if the grammar is recursive. In GE, the chromosome will comprise a maximum number of codons, from which not all may be used during the decoding process. As a result, this turns out to be a natural approach to encoding variable-length solutions.

The next evolutionary operators will be used for the process of grammatical evolution:

- Tournament selection, with tournament size τ. The individual with the highest fitness wins the tournament.
- Single-point crossover: in GE, the reproduction will be performed using only one point for crossover, and will occur with a probability of β. If crossover does not occur, then parents will be present in the following population. Crossover is forced to occur within the subsequences of the chromosomes that are actually used, thus guaranteeing that the crossover has an effective impact in the phenotype.
- Integer-flipping mutation, with a mutation rate of α. The old integer will be replaced by a new random integer. All individuals are mutated, with the sole exception of elite individuals. Mutation also affects the parents that are not crossed and are passed to the next generation. None, one, or more than one positions can be mutated, depending on the value of α.
- Elitism of size *e*. It must be noted that elite individuals are chosen based on the nominal fitness rather than the adjusted fitness.

Regarding the fitness function, we have computed the F1 score of a network trained during five epochs over a validation set using only a 5% sample of the training data. Both the number of epochs and the training set were drastically reduced because of the enormous size of the data set, and the computational requirements that CNNs demand. A complete training of the architectures, defined by individuals of the population, would make the learning process absolutely unfeasible. It was considered that having estimates of the fitness of individuals is a good alternative, and that these estimates—although not entirely accurate—do not necessarily have to introduce any bias that frustrate navigation through more promising areas of the search space. In this way, the evolutionary process becomes viable without being overly hindered by the use of estimates, since the final solutions will be trained with a sufficient number of cycles and with the whole training set.

Finally, we will use niching as a mechanism for preserving the diversity of the population. By doing so, we will prevent the population from converging to very similar individuals. Additionally, by enforcing a certain degree of diversity, we will be able to explore the performance of committees of CNNs, where several models will work together towards performing human activity recognition. In the niching scheme followed in this paper, we have adjusted the fitness value of individual $i_{i}$ as expressed in Equation (3):

(3) $$f_{a}\left(i_{i}\right)=f_{n}\left(i_{i}\right)\times \left(1-\frac{\sum_{i_{j}\in P,j\neq i}sim\left(i_{i},i_{j}\right)}{|P|-1}\right)$$

The similarity function checks how similar two individuals $i_{i}$ and $i_{j}$ are, and is defined in Equation (4):

(4) $$sim\left(i_{i},i_{j}\right)=\left\{\begin{array}{cc} \frac{|i_{i}\cap i_{j}|}{\left|i\right|}, & \mathrm{if}\;n_{c}^{\left(i\right)}=n_{c}^{\left(j\right)}\;\mathrm{and}\;n_{d}^{\left(i\right)}=n_{d}^{\left(j\right)}\\ 0, & \mathrm{otherwise}. \end{array}\right.$$

**Figure 3:**
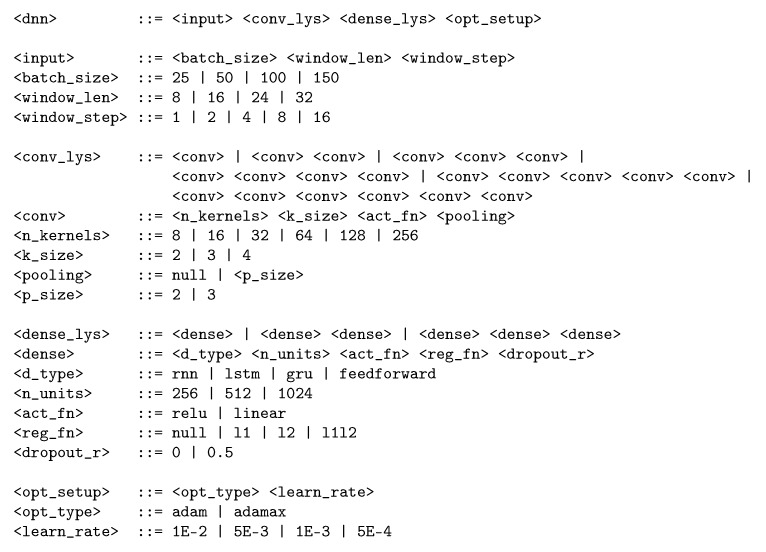
Definition of the grammar in Backus–Naur Form for the OPPORTUNITY dataset.

**Figure 4:**
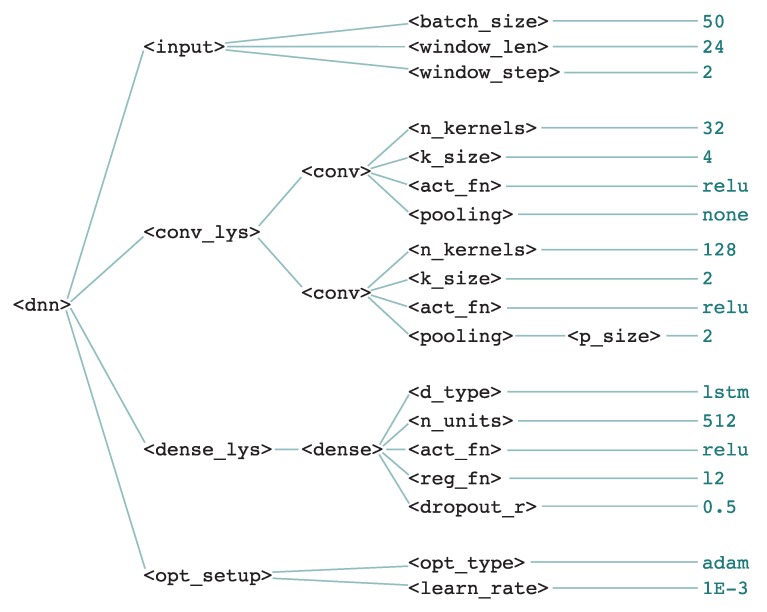
Derivation tree of a sample individual.

Figure 3 shows the definition of grammar used for generating individuals in the OPPORTUNITY problem, in Backus–Naur Form (BNF). Additionally, Figure 4 shows the derivation tree for a sample individual which can be generated by the grammar. This individual comprises two convolutional layers, the first one having 32 kernels of size 4 and the second 128 kernels of size 2, both with ReLU activation, followed by a pooling layer of size 2. Then, the features maps are passed through one recurrent layer with 512 LSTM units with ReLU activation, L2 regularization, and dropout of 50%. The model is trained using the Adam algorithm with a learning rate of $10^{-3}$ using batches of 50 samples, which are obtained from the input data with a sliding window of size 24 and step of 2.

## 6. Evaluation

In this section, we describe the whole evaluation procedure, by first describing the experimental setup for the neuroevolutionary process and later providing an extensive discussion of the obtained results, from the best-performing topologies to the main mistakes occurring during classification with the best model found.

### 6.1. Experimental Setup

The parameters for the grammatical evolution are illustrated in Table 4. The fitness function to be maximized is the F1 score over the validation set of the convolutional neural network whose architecture is defined by the phenotype. F1 score is the main metric reported in the stat-of-the-art for OPPORTUNITY.

As mentioned before, due to the high time consumption requirement of the learning process of the CNNs, we are forced to train the the CNNs using a shortened version of the dataset, largely reducing the learning time. In particular, five epochs were run, with a randomly-chosen 5% of the training set in each of them.

### 6.2. Results and Discussion

Table 5 shows the topology and fitness (F1 score) of the top seven individuals of the hall-of-fame. Symbols “c” and “d” span the convolutional and dense layers, respectively, in order to ease their identification. The best individual had a fitness value of 0.9094, which corresponds to the F1-score resulting from evaluating the CNN when a simplified training process comprising only five epochs and 5% of the training data was used. Additionally, the next individuals performed similarly, all of them with a fitness above 0.9.

Now, when interpreting the phenotypes of these individuals, we can draw some interesting conclusions regarding the CNN topologies they represent. This is specially true if we remember that specific mechanisms such as niching were implemented in order to enhance the genetic diversity and thus consistency between different individuals in the hall-of-fame can be interpreted as a sign that those values performed well.

For example, when considering the input setup, the batch size did not seem to be a critical factor. On the other hand, the sliding window size and step were unanimous across all individuals: a window of 32 samples was always preferred, with a window step of 1. The fact that the minimum step was preferred may be due to sampling: training data was reduced to speedup the experiments, and larger steps would decrease the amount of training data even more by reducing the number of input windows, potentially having a negative impact on effectiveness.

Additionally, at least two convolutional layers are always required: two individuals had two convolutional layers, another two had three layers, one had four layers, and two had five layers. Models with a larger number of layers would be extracting very-high-level features from the raw data in order to increase the classification performance. However, the results show that the network could still perform properly even with less layers. However, one convolutional layer seems insufficient to achieve this task.

Convolutional layers present a very diverse number of kernels, although in all cases there was at least one layer with 128 or 256 convolutional kernels. Small kernel sizes—no larger than four positions—are preferred. Pooling is used in some cases, but there seems not to be a trend regarding its presence in the topology.

Regarding the number of dense layers, all individuals implemented only one of them. This happened because during experimentation, a larger number of layers significantly increased the network complexity and the models did not fit in memory, thus being unable to run and attaining a fitness of 0. Nevertheless, it seems clear that few dense layers can still lead to proper classification.

There is an additional realization at this point: all dense layers were recurrent. This strongly suggests that recurrent layers were required to capture the temporal knowledge present in the input data in this domain. Additionally, vanilla recurrent layers were not used at all, as the models only comprised LSTM or GRU layers, something that can be explained as these cells are more effective and efficient than classical recurrent approaches. The number of hidden units was always either 512 or 1024, a fact that could point out that a smaller number of units was not suitable to properly classify instances. L1 or L2 regularization was never used, and dropout was only found in one case.

All topologies included at least one convolutional layer and one dense layer with a non-linear activation function (ReLU). This may be due to classification being unfeasible when only linear transformations are considered. In this case, regularization was not applied in most models, with the only exception of dropout included for one individual. This could be a sign that overfitting was not happening during the optimization task, even if the training data size was reduced by sampling and despite its large dimensionality comprising 113 channels. As a result, overfitting should not occur either when a larger training dataset is used.

Finally, regarding the learning rule, the choice was in all cases Adam, pointing out that these optimizers are more suitable for achieving an accurate classification. The learning rate oscillated between $\eta =1\times 10^{-3}$ and $\eta =5\times 10^{-4}$.

**Figure 5:**
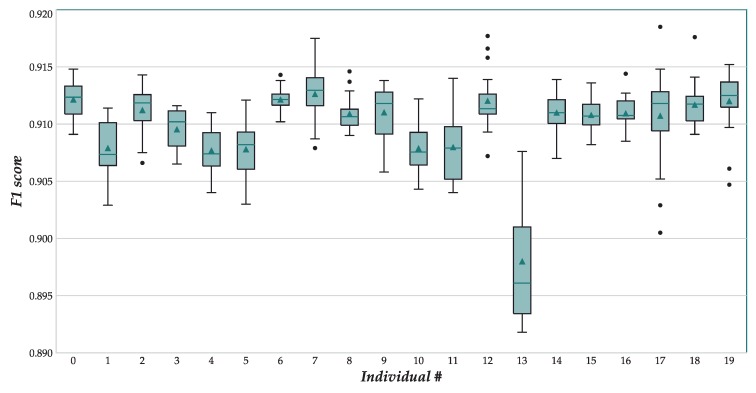
Boxplot showing the distribution of F1 scores of the best 20 GE individuals after full training in OPPORTUNITY Gestures.

Once the neuroevolutionary process suggested a population of good topology designs, we proceeded to perform a full training of each of the top-20 topologies in the hall-of-fame using the entire training set and 30 epochs. The resulting F1 scores are described in Table 6, and depicted in Figure 5. The maximum value reported an F1 score of 0.9185, which is significantly better than the score reported by Ordoñez and Roggen [4] (0.915, see Table 3), but still worse than the best result of the state-of-the-art [5] (0.927).

The comparative results of the two tables—with complete training and without it—confirm the validity of our hypothesis that the use of reduced training as an estimate of the fitness of individuals hardly affects the evolutionary process. The performance values were not very different, and, even better, there was little discrepancy between best and worst individuals in the two tables.

One of the advantages of population algorithms is that they do not generate a single solution, but a set of them. Additionally, a hall of fame mechanism has been included in our model, which stores the best 20 solutions produced throughout the evolutionary process. We used this hall of fame to compose a committee of CNNs. That is, an ensemble of models that can work together to output a single response using a majority-voting policy, therefore guaranteeing that the output class had a certain degree of consensus about the different models.

**Figure 6:**
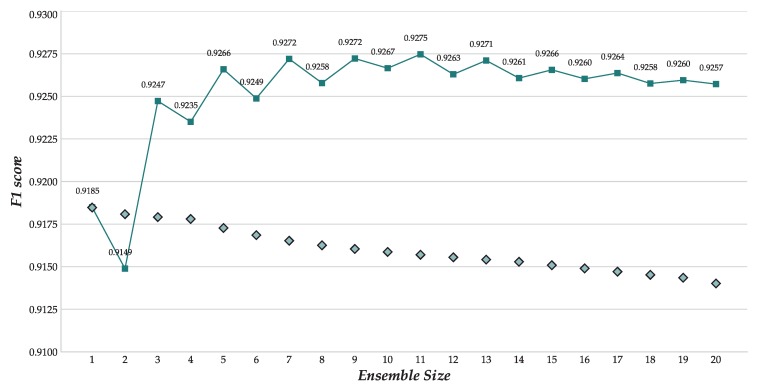
Evolution of the F1 scores of the incremental ensembles using the best 20 individuals from the GE with OPPORTUNITY Gestures.

The performance of these committees as new models were added to them is shown in Figure 6 in the dark line with squares, while the diamonds indicate the average F1 score for all the models included in the ensemble when not acting as a whole. The F1 score showed a large variance with small committees, but stabilized after more than ten CNNs were added to it. The best committee found comprised 11 models, and attained an F1 score of 0.9275. For the first time, this ensemble would slightly exceed the F1 score reported by Hammerla et al. [5] (0.927, see Table 3), therefore heading the ranking.

**Figure 7:**
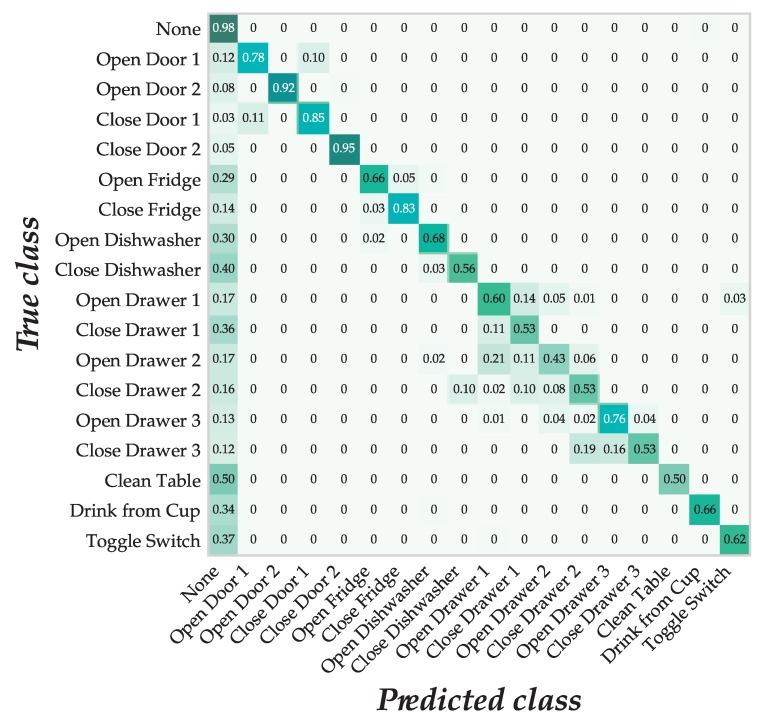
Confusion matrix of the best ensemble using GE individuals with OPPORTUNITY.

Finally, Figure 7 shows the confusion matrix corresponding to the best committee found for the OPPORTUNITY Gestures dataset. Since the classes distribution was unbalanced, we normalized the values per class in order to ease its visualization. Numbers in a row may not sum up to one due to rounding. Most of the activities were, to a greater or lesser degree, misclassified as “null” (no action). This was more frequent in actions related to opening and closing drawers, and especially noticeable for the gestures of toggle switching and cleaning the table. Again, a common confusion seems to involve mixing up opening and closing actions. However, in the current case, this happened mostly with door 1, whereas the second door did not seem affected by this phenomena. Additionally, the system sometimes failed to discern between drawers 1 and 2. In fact, the only activity with a recall lower than 50% was opening the second drawer (43%), and it is remarkable than 21% of the times it was recognized as opening the first drawer. This seems to be a common mistake, easily explainable by the fact that the drawers were located next to each other.

While it is not easy to identify why these actions are poorly recognized, intuition suggests that toggle switching may require very little motion, and therefore the system may fail to recognize that the subject has performed an action at all, whereas cleaning the table may be an activity performed differently across subjects since there were no specific instructions on how to clean the table or how much surface needed to be cleaned. On the other hand, actions related to opening and closing the doors and the fridge showed a higher level of accuracy. It is worth noting that these actions were generally performed very similarly across different subjects.

Aside from actions misclassified as “null”, other mistakes seem easier to explain. In most cases, opening a certain object was sometimes confused with closing it, and vice versa. For example, 78% of the times the gesture “open the door 1” was correctly classified, but 10% of the times it was misclassified as closing door 1. On the other hand, 85% of the times closing door 1 was correctly classified, with 11% of the times being misclassified as opening it.

To different extents, this happened consistently with all objects. However, the confusion was larger in the case of the drawers, where misclassification can involve a different drawer. For example, closing drawer 3 was recognized 19% of the times as closing drawer 2. These mistakes may be acceptable, because the three drawers are located close to each other in the same piece of furniture [45] and the body actions required to open and close them may be similar. Additionally, 10% of the times, closing the dishwasher was mistakenly recognized as closing drawer 2, despite them not being located immediately next to each other.

## 7. Conclusions and Future Work

This work deals with a well-established research challenge when working with sensor networks, which is context-aware activity recognition. In our specific case, we have focused this research on the OPPORTUNITY dataset. This dataset consists of a large number labelled observations coming from a network of heterogeneous sensors (i.e., from wearable, object, and ambient sensors). In this type of scenario where different types of sensors are present, the use of traditional machine learning techniques has important issues, since there are many different types of data inputs collected with usually mixed frequencies and ranges. In addition to heterogeneity, dimensionality (the number of features or inputs introduced into the models) is also another important concern when applying traditional machine learning algorithms.

To tackle these problems (which are intrinsic to most sensor networks), we propose the use of deep learning techniques—more precisely, a technique known as “convolutional neural networks”(CNNs), requiring almost no preprocessing of the input data. However, when working with CNNs, the challenge comes from designing a proper topology for a given sensor network. It is very easy to generate incorrect designs that will return inaccurate results once implemented. Therefore, instead of manually trying different topologies until eventually finding a suitable one, we will let an evolutionary algorithm design optimal topologies.

However, the automatic generation of good (and even valid) CNN architectures is far from being trivial. In fact, the space of possible architectures is virtually unlimited, even when it is reduced to only those that can be considered acceptable. In addition to the fact that the evaluation of each of the alternatives needed to guide the process of evolution is highly time consuming, this makes the process of evolution unfeasible. To solve this problem, the procedure for evaluating the quality of a CNN topology—which consists of checking to what extent the CNN behaves as a good classifier according to the F1 score—has been replaced by a proxy that estimates the CNN performance. This estimate is used as a fitness value, and consists of carrying out the learning with a very limited set of instances (5% of the original training data) and during a very limited time interval (5 training epochs). We will consider that topologies that are able to classify the examples better under these conditions will also behave better in a real-world setting (without sampling and with further training epochs). This hypothesis does not have to be entirely true, but it is enough to discriminate between good and bad architectures, which allows guiding the search process towards better solutions, all the more so as there is no single architecture to solve the problem, but there are a large number of alternatives that behave equally well.

A grammatical evolution method was used for the evolution of the CNNs. The GE has as its core the definition of a grammar in normal form, from which the network designs are generated based on the derivative tree determined by individuals in a population. This mechanism makes it more flexible than other evolutionary methods, and therefore more appropriate, since the designs of the CNNs can be very diverse, with more or less layers, each of which may have more or less parameters.

Given the advantage of having a diverse population of evolved individuals, we decided to perform an extensive training to each of them (using 30 epochs and no sampling of the training data) and to build a committee of neural networks out of them, so that they can work together to decide on a single response when classifying an instance. As a result, we were able to find a classification model capable of achieving an F1 score of 0.9275, outperforming previous results reported in the state-of-the-art, showing how automatically-built evolutionary neural networks can improve those designed by humans, getting simpler and more efficient CNNs for context-aware activity recognition in sensor networks.

The main contribution of this work is that we have shown that it is possible to obtain competitive results when performing human activity recognition in complex environments using deep learning with neuro-evolved topologies. This means that, unlike in many other problems, the topology of the neural networks was designed automatically, without requiring arbitrary hand-made designs (resulting from trial and error) or assistance from experts, which is a remarkable weakness in most deep learning applications. In this work, the optimal design obtained using the OPPORTUNITY dataset might not necessarily work properly if transferred to a problem with a different sensor setup or human activities. In this sense, the availability of a system that is able to automatically infer optimal topologies makes it easier to apply deep learning to a variety of domains and problems.

As future research work, we are interested in exploring the application of our proposal to different datasets involving wider sensor networks or human activity recognition using more pervasive sensor setups, such as those available in smartphones or wearable devices. There is also research interest in studying whether topologies learned for one problem can be transferred to different problems of similar domains where the problem definitions (e.g., the number of channels, the placement of sensors, etc.) are different yet hold some similarities.

## Figures and Tables

**Table 1:** Brief comparison of the features of our neuroevolutionary system with related works. Works marked with a dagger (†) do not use evolutionary computation, but rather reinforcement learning. The comparison criteria include whether the proposal supports a variable number of layers (Var. Ly.), and whether it evolves convolutional layers (Conv.), fully-connected layers (FC), recurrent layers (Rec.) or some of their hyper-parameters, activation functions (Act. Fn.), optimization hyperparameters (Opt. HP), ensembles of neural networks (Ens.) or weights (W). CoSyNE: cooperative synapse neuroevolution; NEAT: neuroevolution of augmenting topologies; CMA-ES: covariance matrix adaption evolution strategy; GA: genetic algorithm; GP: genetic programming; CGP: cartesian genetic programming; RL: reinforcement learning; GE: grammatical evolution.

Work	Technique	Var. Ly.	Conv.	FC	Rec.	Act. Fn.	Opt. HP	Ens.	W
Koutník et al. [19]	CoSyNE								•
Verbancsics and Harguess [20]	GA (NEAT)		•		•				
MENNDL [22]	GA		•						
Loshchilov and Hutter [23]	CMA-ES		•	•			•		
GeNet [25]	GA	•	•						
CoDeepNEAT [26]	GA (NEAT)	•	•	•	•	•	•		
EXACT [27]	GA (NEAT)	•	•						
Real et al. [28]	GA (NEAT)	•	•					•	
DEvol [34]	GP	•	•	•		•			
Suganuma et al. [29]	CGP	•	•						
MetaQNN [30] †	RL	•	•	•				•	
Zoph and Le [31] †	RL	•	•	•	•	•			
This work	GE	•	•	•	•	•	•	•	

**Table 2:** Sensors used in the OPPORTUNITY dataset, placed over the body, the objects, and the environment.

ID	Sensor System	Location and Observation
B1	Commercial wireless microphones	Chest and dominant wrist
B2	Custom Bluetooth acceleration sensors [36]	12 on the body to sense limb movement
B3	Custom motion jacket [37]	Includes 5 commercial RS485-networked XSens inertial measurement units [38]
B4	Custom magnetic relative pos. sensor [39]	Senses distance of hand to body
B5	InertiaCube3 [40] inertial sensor system	One per foot, on the shoe toe box, to sense modes of locomotion
B6	Sun SPOT acceleration sensors	One per foot, right below the outer ankle, to sense modes of locomotion
O1	Custom wireless Bluetooth acceleration and rate of turn sensors	12 objects in the scenario to measure their use
A1	Commercial wired microphone array	Four in each room side to sense ambient sound
A2	Commercial Ubisense localization system	Placed in the corners of the room to sense user location
A3	Axis network cameras	Placed in three locations for localization, documentation, and visual annotation
A4	XSens inertial sensor [37,38]	Placed on the table and the chair to sense vibration and use
A5	USB networked acceleration sensors [41]	8 placed on doors, drawers, shelves, and the lazy chair to sense usage
A6	Reed switches	13 placed on doors, drawers and shelves, to sense usage providing ground truth
A7	Custom power sensors	Connected to coffee machine and bread cutter to sense usage
A8	Custom pressure sensors	3 placed on the table to sense usage after subjects placed plates and cups on them

**Table 3:** Side-by-side comparison of the most relevant results provided in the state-of-the-art for the OPPORTUNITY dataset, including both the locomotion and the gesture recognition tracks, with and without *null* instances. The dagger (†) near some values indicate that performance was reported in a subject-per-subject basis, and are the outcome of averaging the F1 score for subjects 2 and 3. NN: nearest neighbors; SPO: structure preserving oversampling; SVM: support vector machines; LDA: linear discriminant analysis; QDA: quadratic discriminant analysis; NCC: nearest centroid classifier; LSTM: long short-term memory; CNN: convolutional neural network; DNN: deep feed-forward neural network; MV: means and variance; DBN: deep belief network; UP, MI, MU, NU and UT are not technical acronyms but the names given by Chavarriaga et al. [45] to different works based on the name of the institutions participating in the OPPORTUNITY challenge.

Technique	Locomotion		Gestures	
	with *null* class	no *null* class	with *null* class	no *null* class
CStar [45]	0.63	0.87	0.88	0.77
1-NN [45]	0.84	0.85	0.87	0.55
SStar [45]	0.64	0.86	0.86	0.70
3-NN [45]	0.85	0.85	0.85	0.56
NStar [45]	0.61	0.86	0.84	0.65
Integrated Framework [47]	–	0.927 ${}^{†}$	0.821 ${}^{†}$	–
SPO + 1NN + Smooth. [47]	–	0.917 ${}^{†}$	0.811 ${}^{†}$	–
SPO + SVM + Smooth. [47]	–	0.897 ${}^{†}$	0.804 ${}^{†}$	–
SPO + SVM [47]	–	0.885 ${}^{†}$	0.797 ${}^{†}$	–
SVM [47]	–	0.883 ${}^{†}$	0.762 ${}^{†}$	–
SPO + 1NN [47]	–	0.890 ${}^{†}$	0.777 ${}^{†}$	–
1NN [47]	–	0.890 ${}^{†}$	0.705 ${}^{†}$	–
LDA [45]	0.59	0.64	0.69	0.25
UP [45]	0.60	0.84	0.64	0.22
QDA [45]	0.68	0.77	0.53	0.24
NCC [45]	0.54	0.60	0.51	0.19
MI [45]	0.83	0.86	–	–
MU [45]	0.62	0.87	–	–
NU [45]	0.53	0.75	–	–
UT [45]	0.52	0.73	–	–
b-LSTM-S [5]	–	–	**0.927**	–
DeepConvLSTM [4]	**0.895**	**0.930**	0.915	**0.866**
LSTM-S [5]	–	–	0.912	–
LSTM-F [5]	–	–	0.908	–
CNN [5]	–	–	0.894	–
DNN [5]	–	–	0.888	–
Baseline CNN [4]	0.878	0.912	0.883	0.783
CNN + Smooth. [49]	–	–	0.822 ${}^{†}$	–
CNN [49]	–	–	0.818 ${}^{†}$	–
MV + Smooth. [49]	–	–	0.788 ${}^{†}$	–
MV [49]	–	–	0.778 ${}^{†}$	–
DBN [49]	–	–	0.701 ${}^{†}$	–
DBN + Smooth. [49]	–	–	0.700 ${}^{†}$	–

**Table 4:** List of parameters used in grammatical evolution, with their values.

Parameter	Symbol	Value
Population size	|P|	50
Maximum number of generations	*G*	100
Number of generations without improvements (stop condition)	$G_{s}$	30
Codon size		256
Maximum chromosome length		100
Tournament size	τ	3
Crossover rate	β	0.7
Mutation rate	α	0.015
Elite size	*e*	1

**Table 5:** Architecture and fitness of the top seven individuals in the hall-of-fame for GE in the OPPORTUNITY Gestures dataset. GRU: gated recurrent unit; LSTM: long short-term memory; ReLU: rectified linear unit.

#	Fitness	Architecture					
1	0.9094		B=25	w=32	$w_{step}=1$	f=Adam	η=0.001
			$ck_{1}=64$	$cs_{1}=4$	$cp_{1}=1$	$ca_{1}=\mathrm{ReLU}$	
		c |	$ck_{2}=128$	$cs_{2}=3$	$cp_{2}=1$	$ca_{2}=\mathrm{ReLU}$	
			$ck_{3}=16$	$cs_{3}=2$	$cp_{3}=3$	$ca_{3}=\mathrm{ReLU}$	
			$ck_{4}=8$	$cs_{4}=2$	$cp_{4}=1$	$ca_{4}=\mathrm{linear}$	
			$ck_{5}=32$	$cs_{5}=4$	$cp_{5}=2$	$ca_{5}=\mathrm{ReLU}$	
		d |	$dt_{1}=\mathrm{LSTM}$	$dn_{1}=1024$	$dd_{1}=0$	$da_{1}=\mathrm{linear}$	$dr_{1}=\mathrm{none}$
2	0.9037		B=25	w=32	$w_{step}=1$	f=Adam	η=0.001
		c |	$ck_{1}=256$	$cs_{1}=2$	$cp_{1}=1$	$ca_{1}=\mathrm{ReLU}$	
			$ck_{2}=32$	$cs_{2}=4$	$cp_{2}=3$	$ca_{2}=\mathrm{ReLU}$	
		d |	$dt_{1}=\mathrm{GRU}$	$dn_{1}=512$	$dd_{1}=0$	$da_{1}=\mathrm{ReLU}$	$dr_{1}=\mathrm{none}$
3	0.9031		B=50	w=32	$w_{step}=1$	f=Adam	η=0.0005
		c |	$ck_{1}=8$	$cs_{1}=3$	$cp_{1}=1$	$ca_{1}=\mathrm{linear}$	
			$ck_{2}=128$	$cs_{2}=2$	$cp_{2}=1$	$ca_{2}=\mathrm{ReLU}$	
			$ck_{3}=128$	$cs_{3}=3$	$cp_{3}=1$	$ca_{3}=\mathrm{linear}$	
			$ck_{4}=64$	$cs_{4}=4$	$cp_{4}=1$	$ca_{4}=\mathrm{ReLU}$	
		d |	$dt_{1}=\mathrm{GRU}$	$dn_{1}=512$	$dd_{1}=0$	$da_{1}=\mathrm{linear}$	$dr_{1}=\mathrm{none}$
4	0.9025		B=50	w=32	$w_{step}=1$	f=Adam	η=0.001
		c |	$ck_{1}=256$	$cs_{1}=2$	$cp_{1}=1$	$ca_{1}=\mathrm{linear}$	
			$ck_{2}=128$	$cs_{2}=3$	$cp_{2}=1$	$ca_{2}=\mathrm{ReLU}$	
		d |	$dt_{1}=\mathrm{GRU}$	$dn_{1}=512$	$dd_{1}=0.5$	$da_{1}=\mathrm{linear}$	$dr_{1}=\mathrm{none}$
5	0.9013		B=25	w=32	$w_{step}=1$	f=Adam	η=0.0005
		c |	$ck_{1}=16$	$cs_{1}=3$	$cp_{1}=1$	$ca_{1}=\mathrm{ReLU}$	
			$ck_{2}=256$	$cs_{2}=3$	$cp_{2}=3$	$ca_{2}=\mathrm{linear}$	
			$ck_{3}=32$	$cs_{3}=2$	$cp_{3}=1$	$ca_{3}=\mathrm{ReLU}$	
		d |	$dt_{1}=\mathrm{GRU}$	$dn_{1}=512$	$dd_{1}=0$	$da_{1}=\mathrm{ReLU}$	$dr_{1}=\mathrm{none}$
6	0.9013		B=25	w=32	$w_{step}=1$	f=Adam	η=0.0005
		c |	$ck_{1}=16$	$cs_{1}=3$	$cp_{1}=1$	$ca_{1}=\mathrm{ReLU}$	
			$ck_{2}=256$	$cs_{2}=3$	$cp_{2}=3$	$ca_{2}=\mathrm{linear}$	
			$ck_{3}=32$	$cs_{3}=2$	$cp_{3}=1$	$ca_{3}=\mathrm{ReLU}$	
		d |	$dt_{1}=\mathrm{GRU}$	$dn_{1}=512$	$dd_{1}=0$	$da_{1}=\mathrm{ReLU}$	$dr_{1}=\mathrm{none}$
7	0.9010		B=25	w=32	$w_{step}=1$	f=Adam	η=0.001
			$ck_{1}=64$	$cs_{1}=4$	$cp_{1}=1$	$ca_{1}=\mathrm{linear}$	
		c |	$ck_{2}=128$	$cs_{2}=3$	$cp_{2}=1$	$ca_{2}=\mathrm{ReLU}$	
			$ck_{3}=16$	$cs_{3}=2$	$cp_{3}=3$	$ca_{3}=\mathrm{ReLU}$	
			$ck_{4}=8$	$cs_{4}=2$	$cp_{4}=1$	$ca_{4}=\mathrm{linear}$	
			$ck_{5}=32$	$cs_{5}=4$	$cp_{5}=2$	$ca_{5}=\mathrm{ReLU}$	
		d |	$dt_{1}=\mathrm{LSTM}$	$dn_{1}=1024$	$dd_{1}=0$	$da_{1}=\mathrm{linear}$	$dr_{1}=\mathrm{none}$

**Table 6:** Summary of F1 scores of the best 20 GE individuals after full training in the OPPORTUNITY Gestures dataset.

#	Mean	Std. Dev.	Median	Minimum	Maximum
1	0.912150	0.001528	0.91235	0.9091	0.9148
2	0.907910	0.002371	0.90735	0.9029	0.9114
3	0.911230	0.002044	0.91185	0.9066	0.9143
4	0.909540	0.001801	0.91020	0.9065	0.9116
5	0.907695	0.002126	0.90740	0.9040	0.9110
6	0.907805	0.002299	0.90820	0.9030	0.9121
7	0.912155	0.001004	0.91215	0.9102	0.9143
8	0.912635	0.002196	0.91295	0.9079	0.9175
9	0.910900	0.001453	0.91065	0.9090	0.9146
10	0.911030	0.002295	0.91180	0.9058	0.9138
11	0.907885	0.002182	0.90755	0.9043	0.9122
12	0.907995	0.002917	0.90790	0.9040	0.9140
13	0.912040	0.002446	0.91135	0.9072	0.9177
14	0.898005	0.005668	0.89610	0.8918	0.9076
15	0.911005	0.001863	0.91100	0.9070	0.9139
16	0.910800	0.001365	0.91070	0.9082	0.9136
17	0.910945	0.001438	0.91075	0.9085	0.9144
18	0.910730	0.004045	0.91180	0.9005	0.9185
19	0.911695	0.001979	0.91175	0.9091	0.9176
20	0.912015	0.002713	0.91250	0.9047	0.9152

## Abbreviations

The following abbreviations are used in this manuscript:

BNF	Backus-Naur Form
CNN	Convolutional Neural Network
GA	Genetic Algorithm
GE	Grammatical Evolution
GRU	Gated Recurrent Unit
LSTM	Long Short-Term Memory
ReLU	Rectified Linear Unit
